# Expression Profile in Rice Panicle: Insights into Heat Response Mechanism at Reproductive Stage

**DOI:** 10.1371/journal.pone.0049652

**Published:** 2012-11-14

**Authors:** Xianwen Zhang, Jiaping Li, Ailing Liu, Jie Zou, Xiaoyun Zhou, Jianhua Xiang, Wirat Rerksiri, Yan Peng, Xingyao Xiong, Xinbo Chen

**Affiliations:** 1 Key Laboratory for Crop Germplasm Innovation and Utilization of Hunan Province, Hunan Agricultural University, Changsha, China; 2 College of Bioscience and Biotechnology, Hunan Agricultural University, Changsha, China; Beijing Institute of Microbiology and Epidemiology, China

## Abstract

Rice at reproductive stage is more sensitive to environmental changes, and little is known about the mechanism of heat response in rice panicle. Here, using rice microarray, we provided a time course gene expression profile of rice panicle at anther developmental stage 8 after 40°C treatment for 0 min, 20 min, 60 min, 2 h, 4 h, and 8 h. The identified differentially expressed genes were mainly involved in transcriptional regulation, transport, cellular homeostasis, and stress response. The predominant transcription factor gene families responsive to heat stress were Hsf, NAC, AP2/ERF, WRKY, MYB, and C_2_H_2_. KMC analysis discovered the time-dependent gene expression pattern under heat stress. The motif co-occurrence analysis on the promoters of genes from an early up-regulated cluster showed the important roles of GCC box, HSE, ABRE, and CE3 in response to heat stress. The regulation model central to ROS combined with transcriptome and ROS quantification data in rice panicle indicated the great importance to maintain ROS balance and the existence of wide cross-talk in heat response. The present study increased our understanding of the heat response in rice panicle and provided good candidate genes for crop improvement.

## Introduction

Currently, the burden of environmental stresses on crop plants is likely to increase because of global warming, and heat stress is a major abiotic stress limiting plant growth and productivity in many areas of the world [1]. As the staple food for more than half of the world’s population, rice (*Oryza sativa L.*) is the model system for genetic and genomic studies of grasses. It was estimated that rice grain yields decline by 10% for each 1°C increase in minimum temperature during the growing season [2]. The Intergovernmental Panel on Climate Change (IPCC) predicted that only if global mean temperature rises by more than 5.5°C will global food prices increase because of failure of supply to keep full pace with demand [3]. Therefore, the importance to understand the molecular basis of rice heat tolerance is driven by both the interest in basic knowledge and the prospect that such knowledge might provide new strategies for improving heat tolerance in rice.

It’s well known that heat shock directly or indirectly leads to the production of reactive oxygen species (ROS) like H_2_O_2_, which is called oxidative stress [4]. H_2_O_2_ is an early component of the heat-signaling pathway [5], which is required for the activation of small heat shock proteins (sHSP) synthesis as well as the over-production of the ROS scavengers such as catalase, superoxide dismutase and peroxidase [4]. The induction of heat-shock protein (HSP) expression is one of the best-characterized responses to high temperature stress, which is similar mechanism of response to high temperatures in all organisms [6]–[8]. A lot of heat-responsive genes and proteins including Hsps, Hsfs, antioxidant enzymes, various transcription factors and calmodulin have been identified and their functions were well elucidated [9]–[12].

Due to the high correlation between the rice growth at reproductive stage and yield, the great attentions were paid to uncovering the actions of rice reproductive tissues. Expression atlases on reproductive stage and entire life cycle of rice were investigated [13], [14]. Using laser-capture technology, 1,158 pollen mother cells-preferential genes were identified as candidate genes involved in meiotic recombination and meiotic cell cycle control [15]. It was reported that high temperature causes rice male sterility during pollen development [16]. A joint analysis of metabolome and transcriptome was performed to comprehend the impact of high temperature on rice grain filling [17]. It’s an important way to uncover the heat response mechanism using rice varieties differing in heat tolerance. The results from Chen’s research group showed that the heat-tolerant rice species 996 exhibited better anther dehisence and pollen fertility rate than heat-sensitive cultivar 4628 [18], which is consistent with the observations on the other rice species [19]. Chen’s group also found that 996 and 4628 were significantly different in high-temperature tolerance. The seed setting percentage of 996 and 4628 were 66.31% and 36.11%, respectively, under seven days with 37°C/30°C high temperature treatment at reproductive stage [20]. Even so, little is known about the heat response mechanism in rice reproductive tissues. Consequently, understanding the heat response mechanism in rice still remains to be an urgent task, and the reproductive tissues undoubtedly become important targets for the researches on stress response.

So far, as a powerful high throughput technique, microarray has been widely used to systematically investigate the molecular reactions by which plants respond and adapt to complicated environment [21]–[23]. It was also applied to profile the tissue- and stress-specific gene expression [13], [14], [16], [24] as well as transcriptome of rice developing caryopses under high temperature [17].

Here, we reported the time course gene expression profile of young panicle from heat-tolerant rice cultivar 996 from 0 min to 8 h after 40°C treatment using Agilent 44K rice microarray to identify heat responsive genes in rice panicle. The motif co-occurrence analysis on promoters of co-expressed genes and a molecular regulation model central to ROS combined with ROS quantification data revealed the importance of ROS balance and complex cross-talk in response to heat shock in rice panicle.

## Results

### Replicate Analysis

To check the quality of our microarray data, the normalized data were log2 transformed, and Hierachical Clustering (HCL) was carried out using Mev. The results show good correlations between sample replicates with correlation coefficient of more than 0.99 (Table S1).

### Differentially Expressed Genes at All the Time Points

**Figure 1:**
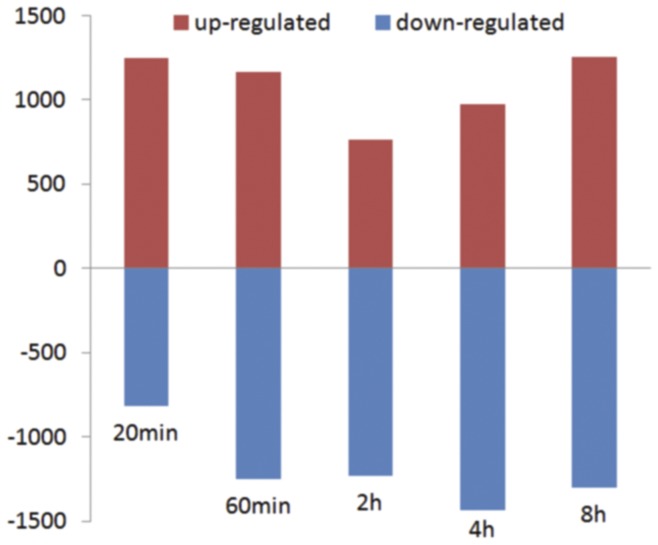
Distribution of the differentially expressed genes at all time points. The numbers on the horizontal axis represented for the time after heat treatment, and that on the vertical axis for the numbers of up- and down-regulated probes.

**Figure 2:**
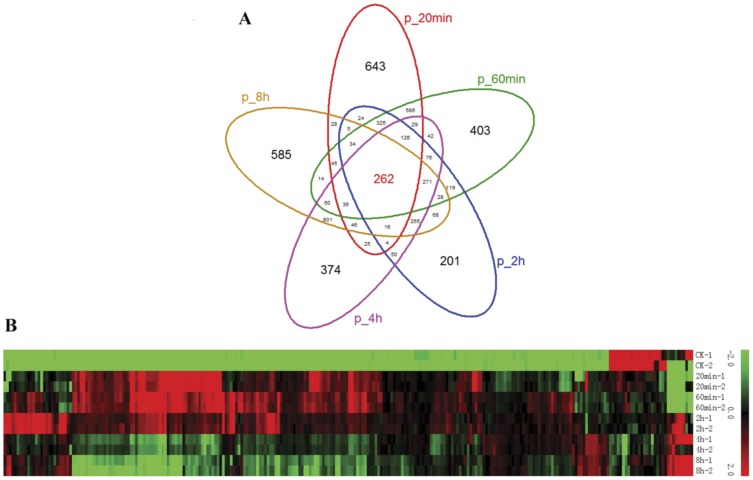
Significantly-regulated probes on the time course of heat stress. A, Venn diagram analysis on the differentially expressed probes at all time points identified 262 significantly regulated probes on the time course of heat stress. B, Expression pattern of the 262 significantly regulated probes using Hierachical Clustering (HCL) algorithm. The color scale (representing the average of normalized values) is shown at the right, and green bar indicates low expression, and red for high expression.

The differentially expressed genes at the time point of 20 min, 60 min, 2 h, 4 h and 8 h heat treatment were determined by more than 3-fold change of expression level compared with the control, and the numbers of up- and down-regulated genes at the five time points are shown in Figure 1. The number of differentially expressed genes at the time point of 2 h heat shock is smaller than that at 20 min and 4 h, therefore, it could be concluded that the time point of 2 h may be the transition and key point for rice panicle in response to high temperature. The Venn diagram analysis shows that there are 198 genes (262 probes) that were significantly-regulated at all the time points of heat stress (Fig. 2A), most of which were early up-regulated (Fig. 2B). These genes are mainly involved in stress-response and transcription regulation according to GO analysis and annotation from RAP-DB, such as zinc finger transcription factors, Hsf, sHsp and ROS-related genes (Table S2).

### Identification of the Heat-responsive Genes

**Figure 3:**
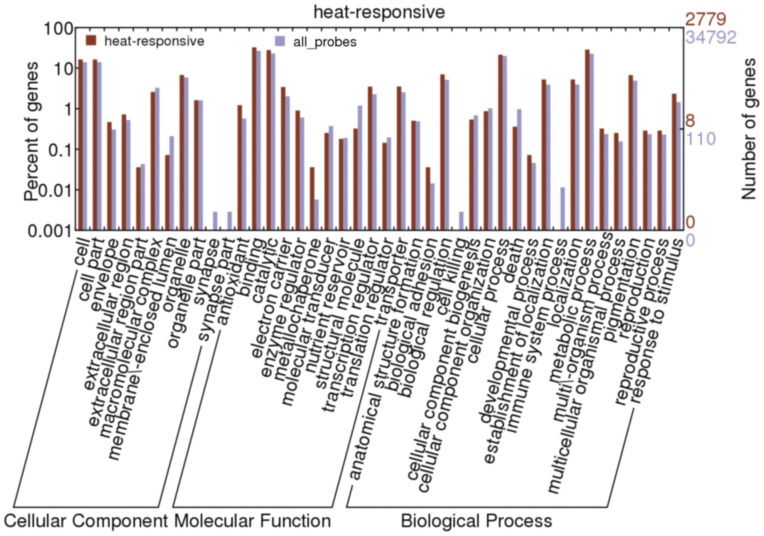
Gene Ontology (GO) classification of the heat-responsive (HR) genes. The HR genes were designated as those have more than 3-fold expression changes compared with the control (untreated sample) in at least two of the five time points.

The heat-responsive (HR) genes were defined as the ones that have more than 3-fold expression change in at least two of the five time points and 2449 genes (3364 probes) were identified as HR genes on the time course (Table S3). Gene Ontology (GO) classification demonstrated that most of these HR genes were related to transporter, protein binding, antioxidant, catalysis, transcription regulation, reproduction and development, stress response categories (Fig. 3).

**Figure 4:**
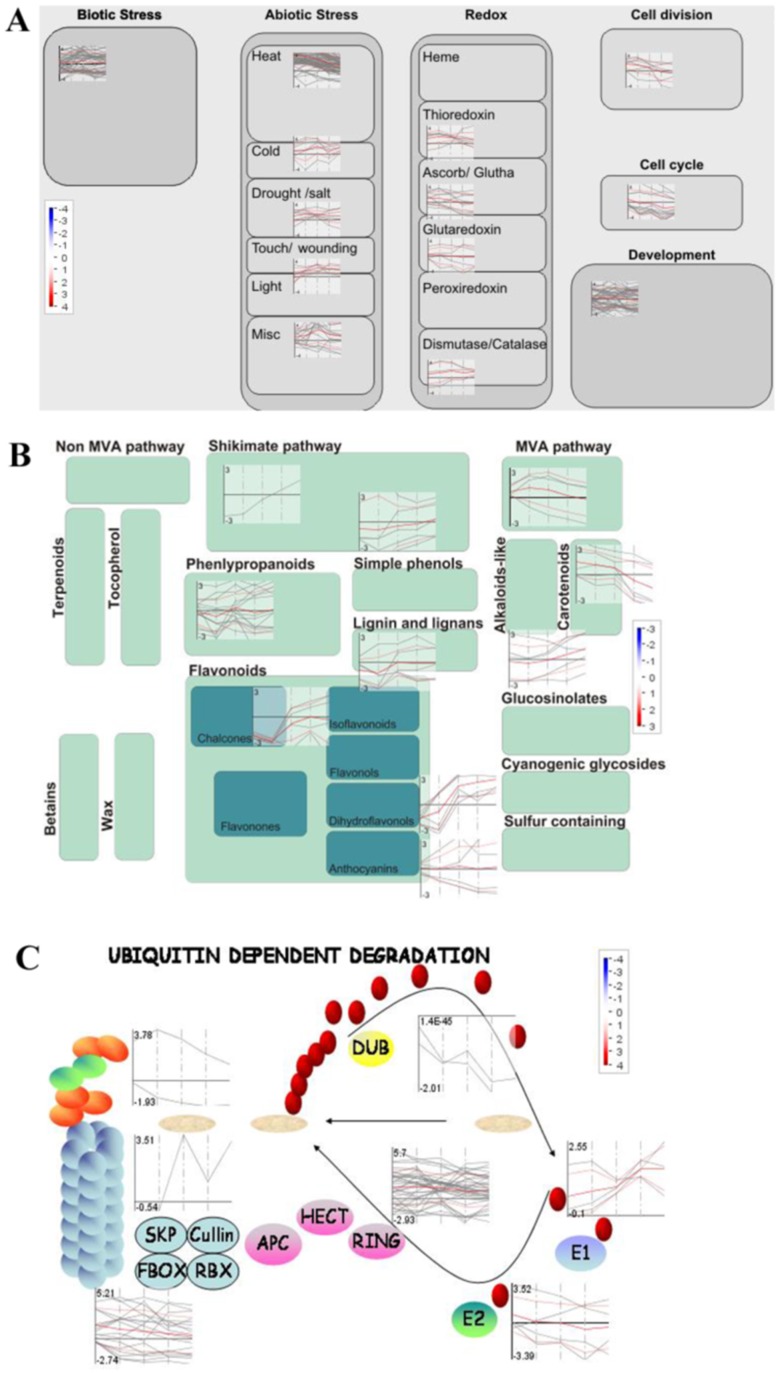
Expression profiles of the genes in cellular response, secondary metabolism and the ubiquitin-proteasome system. MapMan was used to visualize the HR genes in the cellular response (A), secondary metabolism (B) and ubiquitin-proteasome system (C). Each BIN or subBIN is represented as a block with each transcript colored red for up-regulation or blue for down-regulation.

The above GO classifications were further validated with analysis of specific important pathways using MapMan. The cellular response-related genes are mainly involved in biotic stress, heat stress, thioredoxin, cell cycle and development, and they are characteristic of early or constitutive up-regulation during the time course of heat stress such as Hsp family members and dnaJ proteins (Fig. 4A). The secondary metabolism-related genes, which significantly participate in the synthesis of phenylpropanoid, lignin, simple phenols and flavonoids, mainly exhibited late up-regulation under heat stress (Fig. 4B). We further investigated the ubiquitin–proteasome system (UPS) and found that RING/U-box- and SCF-related genes from E3 family constitute a major group with significant and complex expression changes under heat stress (Fig. 4C, Table S9).

### Expression Pattern on the Time Course of Heat Shock

**Figure 5:**
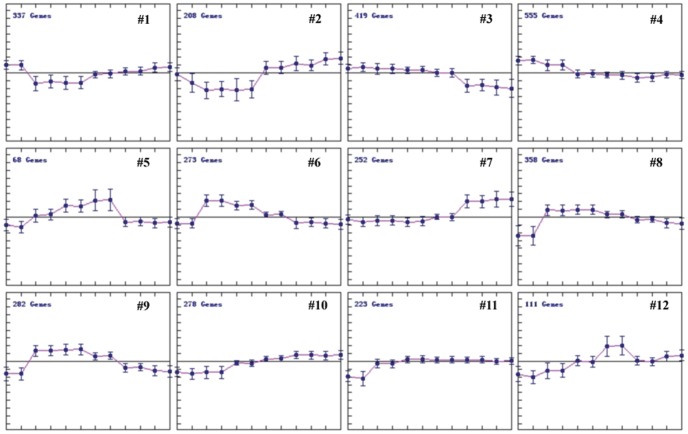
Expression patterns of the HR genes. The HR genes were divided into 12 clusters (from #1 to #12) based on their expression level using k-Means clustering (KMC) algorithm, and #1 belongs to early down-regulated, #2 for early down and late up-regulated, #3 for late down-regulation, #4 for continuous down-regulation, #5, #6, #8 and #9 for early up-regulation, #7, #10 and #11 for late up-regulation, #12 for continuous up-regulation. The vertical axis represented for the gene expression signal value, and the horizontal axis represented for the 12 samples in order (from left to right: CK-1, 2; 20 min-1, 2; 60 min-1, 2; 2 h-1, 2; 4 h-1, 2; 8 h-1, 2 ).

**Figure 6:**
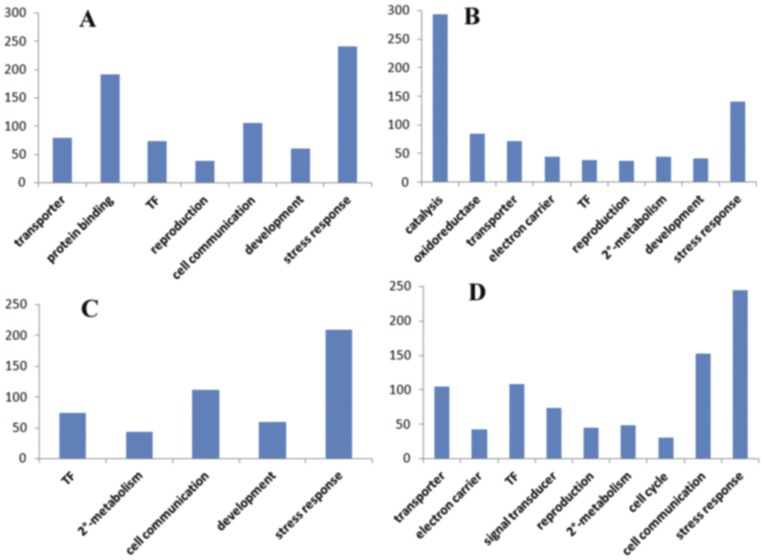
GO analysis on the clusters from KMC. Functional distribution of the early-up- and early-down-regulated (A, B) plus late-up- and late-down-regulated (C, D) clusters showed that the genes are mainly involved in stress response, transcription factor, transporter, secondary metabolism, development as well as reproduction (p = 0.05).

Considering the complex changes of genome-wide gene expression on the time course of heat stress, we used k-Means Clustering (KMC) together with HCL to see the gene expression pattern in rice panicle under heat stress. The HR genes on the time course of heat stress were divided into 12 clusters (Fig. 5). The cluster 1 represents for early down-regulation, cluster 2 for early-down and late-up-regulation, cluster 3 for late down-regulation, cluster 4 for continuous down-regulation, cluster 5, 6, 8 and 9 for early up-regulation, cluster 7, 10 and 11 for late-up-regulation, and cluster 12 for continuous up-regulation. GO analysis on the clusters from KMC showed that the genes involved in protein binding and degradation were early up-regulated, the catalysis-related genes were early down-regulated, and the genes for electron carrier and signal transduction were late down-regulated (p = 0.05) (Fig. 6).

### Major Functional Categories of HR Genes in Rice Panicle

#### Transcription factors

The rice genome has at least 1,930 predicted transcription factors (TFs), which have been classified into 63 families [25], and the rice microarray contains 1,702 TF genes. In this study, the identified TF genes mainly belong to the HSF, AP2/ERF, bHLH, bZIP, MYB, WRKY, NAC and C_2_H_2_ families (Table S4). Among the 9 heat responsive *Hsf* genes, 5 belong to *HsfA* sub-family (*HsfA2a*, *HsfA2d*, *HsfA9*, *OsHsfA3*, *OsHsfA2f*) and 3 belong to *HsfB* sub-family (*HsfB2a*, *HsfB2b*, *HsfB2c*). They exhibited early-up- or constitutive-up-regulation during the period of heat treatment. Notably, the transcript for *HsfA2a* and *HsfA2f* were induced over 200-fold at the beginning of heat shock. Twenty-four of the 164 *AP2/ERF* TF genes were identified as HR, and only one *AP2* and 5 *DREB* sub-family members were significantly expressed. The *ERF* subfamilies, *B1*, *B2*, *B3* and *B4*, constitute the major groups. Among them, Os01g0313300, Os03g0182800 and Os02g0546800 showed continuous up-regulation upon heat stress. Among the 9 HR genes in the 178 *bHLH* members, only Os08g0487700 was early up-regulated, and all the others were repressed during heat stress. Among the 13 HR *bZIP* family genes, *OsbZIP-IV* and *VI* subfamilies were major groups. Only 3 genes from *OsbZIP-VI*, 2 from *OsbZIP-III* and 1 from *OsbZIP-I* were early up-regulated. In addition, among the *Myb* family genes, 9 genes for (R1)R2R3, 7 for MYB-related and 3 for atypical MYB were identified as HR. The 11 identified HR *WRKY* family genes were mainly from group II and III. Only 3 genes (Os05g0537100, Os11g0117400 and Os12g0116600) were continuously up-regulated during heat shock, and the others were characteristic of early induction. There are 13 HR genes from *NAC* family, and *SNAC_B* subfamily is predominant. Similarly, among the 11 HR genes in *C_2_H_2_* family, a large part of them were early induced in rice panicle under heat shock.

#### Hsp and other chaperone genes

It is well known that plant HSPs and other chaperones play important roles both in response to adverse environmental conditions and in various developmental processes. Our experiments identified 39 Hsp genes and 33 other unclassified Hsp genes responsive to heat (Table S5). Among the HR Hsp genes, 3 genes represented for Hsp100, 11 for Hsp70, 6 for Hsp90, and the other 19 genes for sHsp. All HR members from Hsp100 family and most HR genes from Hsp70 and Hsp90 families exhibited significant elevation at the transcript level within 4 h after heat treatment in rice panicle. Only 4 genes for Hsp70 and 2 genes for Hsp90 were persistently induced by heat. Notably, almost all of the HR sHsp subfamily genes were greatly and continuously up-regulated during the period of high temperature, and constituted a major group of the significantly-regulated genes on the time course of heat treatment (Table S2, Table S5). Most of the unclassified HR Hsp genes were early induced. A few genes for DnaJ protein, dehydrin, and hypothetical proteins were persistently elevated more than 10-fold during heating in rice panicle.

#### Transporters

In the rice genome, transporter family genes are grouped into 4 distinct types: ATP-dependent transporters, secondary transporters, ion channels, and unclassified transporters. Among 1,286 transporter-encoding genes, 1,147 genes were present in rice microarray, and 115 were significantly altered at the transcript level under heat treatment and considered as HR genes (Table S6). Among the 8 ATP-binding cassette (ABC) transporter genes responsive to heat, 2 genes (Os09g0572400 and Os03g0332700) responded to early heat stress, 2 genes (Os03g0281900 and Os12g0132800) exhibited early repression and late induction. The rest 4 genes were significantly down-regulated. Among the genes related to sugar, peptide, amino acid and other metabolites transport, 7 genes were remarkably early heat-induced. At the same time, 8 genes for aquaporin were identified as HR genes. Only *OsNIP2;1* (Os02g0745100) and *OsTIP1;2* (Os01g0975900) were continuously up-regulated during heating in rice panicle, and both of *OsNIP4;1* (Os01g0112400) and *OsTIP4;1* (Os05g0231700) were late elevated. The other 4 genes including *OsPIP2;4* (Os07g0448100) were repressed.

#### ROS-related genes

ROS-related antioxidant enzyme systems mainly include superoxide dismutase (SOD), catalase (CAT), ascorbate peroxidase (APX), peroxidase (Prx), glutathione peroxidase (GPX), glutathione-S-transferase (GST), alternative oxidase (AOX) and peroxiredoxin (PrxR), and they have pivotal roles in ROS clearance and balance. In the present report, 225 ROS-related genes were detected among HR genes, and mainly comprised P450 family, GST, Prx and thioredoxin (Trx) (Table S7). Most of the P450 family HR genes were repressed to some extent, and only 10 P450 family genes were up-regulated at the late stage of heat stress in rice panicle. Most of the Prx genes were repressed during heat stress, and just a few were late induced. All HR Trx genes were early induced. Notably, the genes for *AOX1b* (Os04g0600300) and PrxR gene (Os07g0638300) were up-regulated more than one hundred-fold at the early stage of heat stress, and remained at high level at the late stage. One gene (Os07g0665200) for SOD was slightly induced during heat treatment.

#### Signal transduction

In our microarray data, 55 phytohormone-related genes were detected as HR genes, and they are mainly involved in auxin, ethylene and ABA metabolism and signaling (Table S8). Among 24 auxin-related HR genes, most were down-regulated under heat treatment, and the 6 up-regulated genes encoded ripening-associated protein (Os03g0796000), SAUR-like protein (Os08g0118500, Os02g0643800 and Os06g0714300), nitrilase (Os02g0635200) and auxin efflux carrier (Os01g0802700), respectively. One SAUR family gene (Os09g0545300) remained at high transcript level during heating. Three ABA signaling-related genes encoding bZIP proteins were early up-regulated. And only one gene for *SAPK9* (Os12g0586100) was late induced under high temperature. Among the 9 HR genes involved in ethylene signaling, three were continuously induced more than 2-fold under heat shock (Os02g0574800 for EIN3, Os06g0592500 for ERF and Os09g0570800).

At the same time, six light-responsive genes were identified as HR, and one encoding a light regulated Lir1 family protein was up-regulated over 2-fold after 2 h of heat treatment, three for early light-induced protein (ELIP) and one for BTB domain containing protein were all early induced upon heat shock (Table S8).

Furthermore, downstream events of phytohormone and other signal molecules would be mediated through various protein kinases. The HR protein kinases mainly comprise LRR family, G-proteins, DUF26 family, calcium-related genes and other kinases (Table S8). Overall, most of the genes from LRR and DUF26 were down-regulated. In contrast, most of calcium-related and G-protein genes exhibited higher expression level. Several genes encoding calmodulin binding protein (Os01g0134700, Os03g0436300, Os06g0256300 and Os12g0547600), Ca^2+^-ATPase isoform 9 (Os08g0517200) and EF hand domain containing protein (Os08g0558100) exhibited early up-regulation. A calmodulin gene (Os01g0810300) was continuously up-regulated. In addition, some genes from the small GTPase family were early up-regulated such as Ras (Os12g0631100), RhoGAP (Os07g0486500), Rab-21 (Os05g0341600) and Ran-2 (Os01g0611100).

### Promoter Analysis of Co-expressed Genes

**Figure 7:**
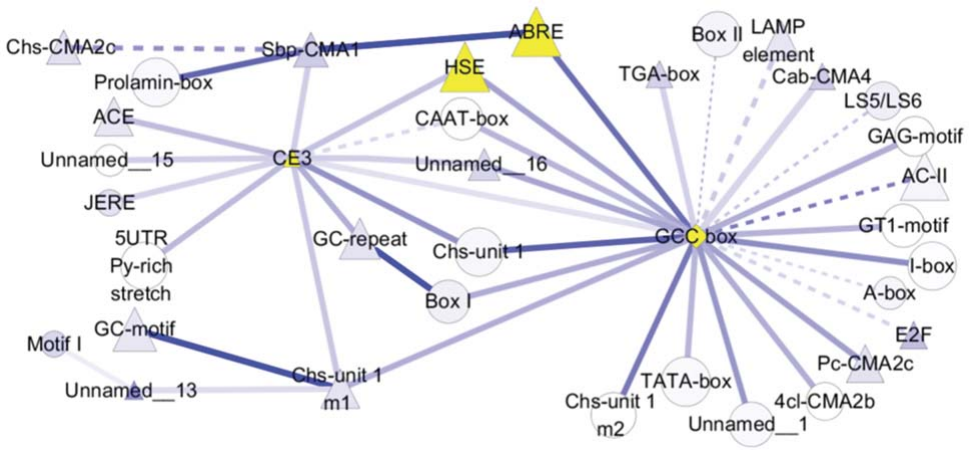
Co-occurrence analysis on the promoters of the HR genes from Cluster 9 of KMC. The motifs were scanned using FIMO in the −3000 bp upstream ATG of all genes in cluster 9. The motif enrichment and significance of motifs co-occurrence were assessed by the hypergeometric distribution, and the data were imported into Cytoscape. The important stress-responsive motifs such as GCC box, CE3, ABRE and HSE were significantly enriched (marked by yellow). Diamond represents the nodes most significantly enriched (p<0.001), triangle for significantly enriched (0.001<p<0.05), and circle for not significantly (0.05<p). Solid lines are referred as the p-value of co-occurrence less than 0.01, and dash lines as p-value between 0.01 and 0.05.

To get more understanding of the gene expression regulation under heat shock, the promoter motifs were scanned using FIMO in the −3000−bp upstream ATG of all genes in rice. The enrichment of the motif in clusters from KMC and significance of co-occurring motifs were assessed by the hypergeometric distribution. Then the data were imported into Cytoscape (Fig. 7). The results indicated that, only in the early up-regulated Cluster 9, the important stress-responsive motifs including GCC box, CE3, ABRE, HSE, GC-repeat and Box I were significantly enriched. The enriched elements constituted two co-occurrence networks, and GCC box and CE3 were the centers of the two networks, respectively. HSE exhibited close relationship with both core elements, but ABRE only with GCC box. In addition, GC-repeat and Box I co-occurred significantly, and they showed close relationship with GCC box and CE3.

### Quantitative Real-time PCR (qRT-PCR) to Confirm Microarray Data

**Figure 8:**
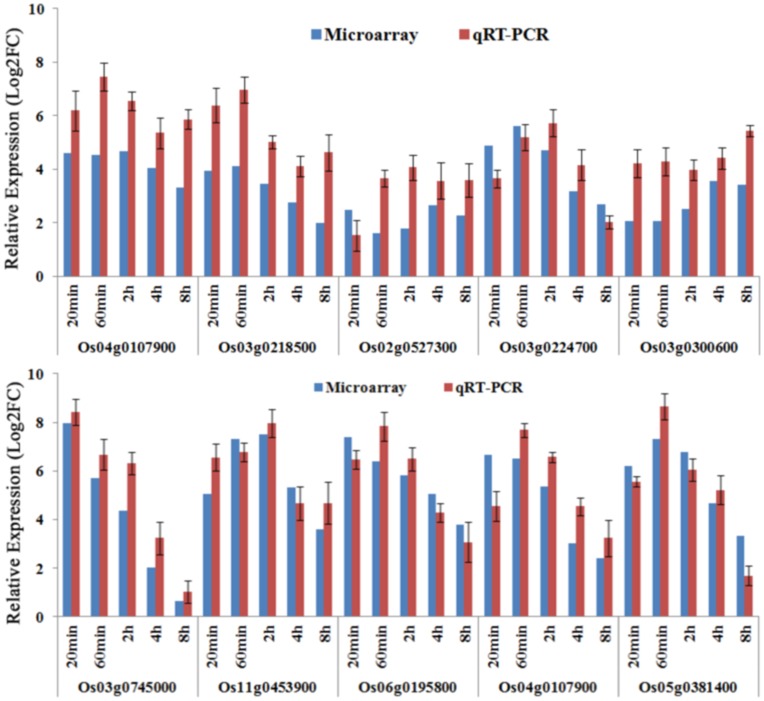
Quantitative Real-Time PCR verification of the genes expression level. Transcript levels were presented as relative values that were normalized with respect to the level of actin 1 gene. Error bars in the figures indicate standard deviation. Log2FC, log2 transformed fold change.

To validate microarray data and investigate the dynamic profile of gene expression, expression level of the 10 genes significantly-altered during heat stress was analyzed by qRT-PCR. The results showed that the expression patterns of all candidate genes by qRT-PCR were consistent with the microarray data (Fig. 8).

## Discussion

### Transcriptional Regulation may Play Vital Roles in Rice Panicle Response to Heat

As triggers for gene expression, TFs play important regulatory roles in almost all aspects of plant life including growth, development, and responses to adverse environmental conditions such as high temperature, drought, high salinity, flood and pathogen attack. In the present study, we identified 8 HR TF families that include *Hsf*, *AP2/ERF*, *bHLH*, *bZIP*, *Myb*, *WRKY*, *NAC* and *C_2_H_2_*.

There are 25 *Hsf* members in rice genome, 21 *Hsf* genes in rice microarray, and they were divided into three conserved classes, A, B and C, based on sequence homology and domain architecture [9]. Our data demonstrated that *HsfA2a*, *HsfA2d*, *HsfA2f*, *HsfA3* and *HsfB2a*, *b* and *c* were remarkably induced within 4 h after heat treatment in rice panicle (Table S4). The genome-wide expression analysis showed that *HsfA2a* and *HsfA2d* were induced over thousand-fold upon heat shock in rice root and shoot, and exhibited high expression level at all stages of panicle development, and that the three *HsfB2* family members, a, b and c, were greatly induced under heat shock in roots, shoot and panicle. It was also demonstrated that *HsfA2f* and *HsfA3* were induced upon heat shock in rice panicle [26]. The previous work in our lab showed that *HsfA2a* and *HsfA2d* were also quickly induced by heat treatment in rice seedling [27]. Further investigation uncovered that *HsfA2* is indispensable for acquired thermotolerance in *Arabidopsis* [28]. These data implied the important functions of the HR genes from *HsfA2* and *HsfB2* sub-families to activate cellular protection system in reproductive development and heat response in rice panicle.

As one of the largest TF families, *AP2/ERF* gene family can be classified broadly into four subfamilies: *AP2*, *RAV*, *DREB* and *ERF* [29]. Previous microarray analysis showed that a *DREB_A1* gene was continuously up-regulated under various virus infections, and an *ERF_B3* was significantly induced by drought [30]. In our case, the first gene was induced only at the beginning of heat shock, and the latter one was late up-regulated during heat stress (Table S4). Tissue expression profile elucidated that one *DREB* gene and three *ERF* genes were greatly repressed in rice panicle under normal condition [29], and they were greatly induced by heat in our research (Table S4), which may suggest they play important roles in rice panicle in response to heat shock. Therefore, *ERF* and *DREB* sub-family genes may be good candidates for improving crop abiotic stress tolerance [31].

The *OsbHLH* family in rice genome has 178 genes [32], and there are 170 *OsbHLH* genes in rice microarray. The previous work revealed that *OsbHLH148* (Os03g0741100) was induced under the treatment of dehydration, high salinity, low temperature and wounding in rice [33], and that *OrbHLH2* from wild rice (*Oryza rufipogon*) may positively regulate salt-stress signals independent of ABA in *Arabidopsis* [34]. Among the 9 HR *bHLH* genes in our data, only Os08g0487700 was early up-regulated during heat stress, and the rest were down-regulated to different extent (Table S4), which implied that *bHLH* family genes may be mainly involved in negative regulation of heat response in rice panicle.

In the genome-wide expression analysis on the *OsbZIP* genes, only *OsbZIP45* was strongly induced by dehydration, salt and cold, and *OsbZIP12*, *OsbZIP60* and *OsbZIP66* were induced by both dehydration and salt, but not by cold. Nevertheless, *OsbZIP45* was up-regulated at one or more stages of panicle development [35]. The bZIP transcription factor, *OsABI5* isolated from rice panicle, could regulate the adaptive stress response and plant fertility [36]. In the present study, 13 HR *bZIP* genes were identified, and 9 of them belong to group III, IV and VI (Table S4). The 2 genes (*OsbZIP50*, *OsbZIP60*) from group III and 4 genes (*OsbZIP45*, *OsbZIP12*, *OsbZIP60* and *OsbZIP66*) from group IV and VI were early induced under heat stress in rice panicle. The data suggested the *OsbZIP* family genes may participate in cross-talk in stress responses.

The overexpression of a salt-inducible gene, *TaMYB32*, enhanced the tolerance to salt stress in transgenic *Arabidopsis* [37]. MYBS3 activated cold signaling pathway in rice [38]. However, MYBC1 negatively regulated the freezing tolerance of *Arabidopsis* [39]. Our data showed that, among 24 HR *Myb* genes, 10 were early elevated, only Os02g0114800 and Os07g0137000 were late up-regulated during heating, and the rest were repressed (Table S4). The recent report demonstrated that *OsWRKY24* and *OsWRKY28* were strongly induced by water-deficit, cold and salt, but *OsWRKY82* was greatly down-regulated [40]. Other reports illustrated the up-regulation of *OsWRKY24* by ABA in aleurone cells [41], [42], *OsWRKY12* in response to SA, JA, wounding [43] and *OsWRKY11* in heat and drought stress response [44]. Our data showed that, among 11 HR *WRKY* genes, *OsWRKY12*, *OsWRKY24*, *OsWRKY28* and *OsWRKY82* were early induced, and only *OsWRKY07*, *OsWRKY56* and *OsWRKY100* were continuously elevated during heat shock (Table S4). Our comprehensive expression analysis would be helpful for deciphering important roles of *Myb* and *WRKY* family genes in heat response in rice panicle.

In the present study, 13 *NAC* family genes were identified as HR. Recent researches revealed that SNAC factors can be useful for improving stress tolerance in transgenic plants [45], an ENAC transcription factor was early induced by abiotic stresses in rice [46], and *OsNAC5* can enhance tolerance to drought stress by up-regulating the expression of stress-inducible rice genes [47]. The 189 *C_2_H_2_* zinc-finger protein (ZFP) genes in rice are also called ZOS (ZPTs of *Oryza sativa*). In the genome-wide expression study [48], *ZOS5-06* (Os05g0279400) gene had a high signal value in all tested tissues including 6 panicle development stages (P1–P6) and was also up-regulated during dehydration stress, and only *ZOS3-21* (Os03g0820300) was up-regulated under all the stresses of cold, drought and salt. Meanwhile, *ZOS5-11* (Os05g0495100) was up-regulated in P1, P2. The present study demonstrated that *ZOS5-06* and *ZOS4-02* were early induced upon heat stress in rice panicle, and only *ZOS3-21* was induced in the consecutive stages of heat shock (Table S4). Taken together, these *NAC* and *C_2_H_2_* family genes may play important roles in rice panicle development under heat.

### The Roles of Hsp and Other Chaperones in Heat Response

Hsps generally function as molecular chaperones, and are divided into Hsp100, Hsp90, Hsp70, Hsp60, Hsp40 and Hsp20 or small heat shock proteins (sHsps). The induction of heat shock proteins when plants are exposed to elevated temperature has been well documented. Our results revealed that chaperone genes constitute the largest family responsive to heat (Table S5).

The analysis showed that Hsp100 proteins are responsive to heat stress in plants [49]. Previous reports proved that *Hsp101* is primarily a high-temperature tolerance mechanism in *Arabidopsis* [50] and reached at maximal mRNA levels under 45°C for 2 h [51]. In the present study, all the HR Hsp100 genes exhibited early induction during heating (Table S5). Therefore, the Hsp100 family genes may play important positive roles in heat stress response among plant kingdom.

The protective HSP70 and HSP90 had increased levels of protein and gene expression following heat treatment [11]. *OsHsp90* from rice maintained *E. coli* growth well at high temperatures [52]. In contrast, overexpressing *AtHsp90.3* impaired plant tolerance to heat stress, which suggested that proper homeostasis of Hsp90 be critical for cellular stress response and/or tolerance in plants [53]. Our data showed that, among the 6 HR Hsp90 family genes, only Os04g0107900 was continuously induced under heat shock, the others were early elevated. It indicated the HR Hsp90 genes were mainly involved in early heat response in rice panicle.

Hsp70 confers tolerance to heat and other abiotic stresses in *Arabidopsis* [54]. The investigation suggested that mitochondrial Hsp70 may suppress programmed cell death in rice protoplasts by inhibiting the amplification of ROS [55]. Earlier studies showed that Hsp70 protein interacts with unfolded proteins [56] and is crucial for the survival of bacteria, yeast and plants under stress conditions [57]. In our case, most of the HR Hsp70 genes were greatly induced at the early stage of heat stress (Table S5), which is consistent with other reseach [58], and three genes remained at high transcript level during heating. Thus, the Hsp70 genes play an important part in response to heat stress in rice panicle.

Among 23 sHsp genes in rice genome [59] that are all present in rice microarray, 19 HR sHsp genes were identified (Table S5). OsHSP26 plays an important role in the protection of PSII during heat (42°C) [60]. Sarkar et al. reported that 19 sHsp genes were induced by high temperature in rice [59]. Constitutive expression of *RcHSP17.8* from *Rosa chinensis* confers wide resistance to heat, salt, osmotic and drought stresses in *Arabidopsis thaliana* [61]. Another group found that sHSP proteins have a non-redundant function in acquired thermotolerance in hypocotyl elongation [62]. Our results showed that most of the sHsp genes were remarkably and continuously elevated during heat shock, and constitute the major Hsp sub-family responsive to heat (Table S5). Therefore, sHsp genes may play a big part in response to heat stress in rice panicle.

The members from DnaJ cochaperone family played important roles in the response to heat and other stresses [63]. DnaJ proteins were involved in correct folding of proteins under stress conditions [64]. The interaction between Hsp70 and unfolded proteins was regulated by cochaperones including DnaJ proteins [65]. In our case, the HR genes for DnaJ or DnaJ -like proteins were significantly altered under heat stress, and most of them were early up-regulated (Table S5). The data demonstrated the importance of DnaJ protein in the heat tolerance in rice panicle.

### Significant Gene Expression Changes to Maintain the Cellular Homeostasis

It’s well known that ROS are rapidly produced upon various stresses in plants, and also lead to series of downstream events such as protein activation, lipid oxidation, and further establishment of new gene expression pattern [66]. Earlier works elucidated that plant Prxs gave rise to ROS production to actively participate in defense reaction [67]. Our data showed that most of Prx genes were down-regulated, and only a few genes were late up-regulated during heat treatment (Table S6), which suggested Prxs may not be the major source of ROS production at the early stage and come into action in the late period of heat shock.

ROS are often important signal molecules to activate ROS-scavenging enzyme system, which is the key to maintain cellular homeostasis. Analysis on two japonica rice genotypes revealed that increased cold tolerance was related to the higher constitutive SOD, APX and CAT expression [68]. Overexpression of the SOD and APX genes improves seed longevity and germination under various environmental stresses [12]. *NtAOX1a* from *Nicotiana tabacum* is necessary for plants to survive oxidative stress [69]. The progress on P450s in plant development and reproduction was reviewed [70], and the roles of P450 in ROS handling and utilization were discussed [71]. Our transcriptomic analysis revealed that the above HR genes constitute the major group involved in ROS-scavenging and signaling (Table S6), and may be important in response to heat shock in rice panicle.

Transporters are usually located in the membrane, and play important roles in metabolism, signal transduction, defense and development. ABC-transporters utilize the energy of ATP hydrolysis to carry out certain biological processes across membranes [72], and are involved in the transport of ABA in response to stresses in *Arabidopsis* [73]. In our case, the genes from ABC family and involved in iron and metabolites transport showed diverse expression pattern during high temperature (Table S6), especially most of them were down-regulated, which may imply some physiological and biochemical processes of transporting be slowed down upon heat in rice panicle.

Moreover, a comprehensive expression analysis in rice identified 33 aquaporin genes, and demonstrated that *OsNIP2;1*, *OsNIP4;1* and *OsTIP1;2* had low expression level in anther, only *OsTIP4;1* had high mRNA level in anther [74]. In our data, *OsNIP2;1* and *OsTIP1;2* were continuously up-regulated during heat shock in rice panicle, and *OsNIP4;1* and *OsTIP4;1* were late induced (Table S6). The low background and highly heat-induced expression suggested the positive roles of both *OsNIP2;1* and *OsTIP1;2* genes in rice panicle under heat stress.

In plants, the ubiquitination of a target protein requiring the sequential actions of the E1, E2 and E3 enzymes from the UPS is emerging as a significant regulatory system to result in specific protein degradation. The roles of the E3 ubiquitin ligase in plant growth, development and stress responses have been reviewed [75]. SIZ1, a SUMO E3 ligase, regulated rice anther dehiscence [76] and in responses to high and low temperature [77]. The early analysis revealed that at least 43 F-box genes of E3 ligase family were differentially expressed in rice seedlings under abiotic stresses [78]. The advances of the UPS in coping with stresses to maintain cellular homeostasis were highlighted [79]. In the present study, among 123 HR genes from the UPS, 58 E3 ligase genes constituted the major group in the UPS (Fig. 4C). Most E3 ligase genes were early or late up-regulated, and only 5 genes were continuously induced, and 11 were repressed during heat shock (Table S9). The data demonstrated the important roles of specific protein degradation at different stage of heat shock. We observed that an *FtsH* gene (Os06g0229000) was continuously induced during heating, which is consistent with the previous finding that FtsH11 protease plays a critical role in *Arabidopsis* thermotolerance [80]. Therefore, it suggested that the HR UPS genes may be vital to rice panicle development and heat response.

Taken together, it is concluded that significant expression changes of genes for ROS-scavenger, transporter, and UPS have important roles in maintaining the cellular homeostasis to cope with heat stress in rice panicle.

### Heat Signal Transduction Facilitates Re-establishing Cellular Balance

It’s a great task to uncover the mechanism of stress signal transduction. We found that, in rice panicle, heat stress signal transduction system consists of the genes encoding calcium-related protein, receptor kinase, G-protein, and light signal-related protein as well as the genes for phytohormone signaling and metabolism.

It was found that the application of auxin can reverse the male sterility caused by high temperature stress in barley and *Arabidopsis* [81]. Nitrilase plays important roles in maintaining auxin homeostasis through hydrolyzing IAA [82]. SAUR39 from the small auxin-up RNA (SAUR) family was characteristic of negative regulator for auxin synthesis and transport, and thus resulted in lower shoot and lower yield [83]. In our case, the induction of one nitrilase and four SAUR genes suggested the regulation of auxin and the delay of rice panicle development under high temperature (Table S8).

ABA signaling pathway has been primarily established [84]. It was uncovered that the relatively high concentrations of ethylene and ABA in inferior spikelets lead to a low grain-filling rate [85]. Ten SnRK2 genes of ABA signaling pathway in the rice genome were identified and designated SAPK1 through SAPK10 that were activated in response to hyperosmotic stress and ABA [86]. Besides, phospholipase D (PLD) and its product phosphatidic acid (PA) mediate the signaling of various plant hormones. PLDβ1 stimulates ABA signaling by activating SAPK, thus inhibiting seed germination [87], and also functions as a negative regulator in salt response in rice [88]. In the present study, three ABA-related *bZIP* genes and one *SAPK9* were early and late up-regulated, respectively. And all HR PLD-encoding genes were repressed during heating (Table S8). Our data verified important roles of the *bZIP* and *SAPK9* as well as PLD genes in rice panicle under heat shock via ABA signaling pathway.

*EIN3* was found as the key gene in the response to ethylene in plant [89]. The *ERF* gene family was identified as one group of the major HR transcription factors (Table S4), and may play vital roles in response to heat stress. In addition, our data showed that an *ERF* coactivator gene (Os06g0592500) was continuously induced under heat shock (Table S8). Therefore, the genes involved in ethylene signaling including *EIN3*, *ERF* and *ERF* coactivator gene may actively participate in heat response in rice panicle via stimulating ethylene-mediated pathway.

Generally, high temperature is accompanied by high light, and so perception of light plays a very important role in heat response. Earlier studies showed that ELIP is correlated with light stress [90] and heat shock [91]. *ELIPs* were induced under salt and wounding treatments [92]. In addition, *Lir1* gene in rice was controlled by light [93]. In our case, the early-induction of the three *ELIP* genes and the great up-regulation of *Lir1* gene after 2 h of heat shock implied their important functions in heat shock response in rice panicle (Table S8).

A proteomic analysis suggested the possible involvement of CaM in resistance to Mungbean Yellow Mosaic India Virus in Vigna mungo [94]. AtCaM3 was found a key component with positive function in the Ca^2+^-CaM heat shock signal transduction pathway in *Arabidopsis* [95]. A stress-responsive Ca^2+^-ATPase gene is an essential component for abiotic stress adaptation in *Physcomitrella* [96]. Due to remarkable up-regulation of the genes for the calmodulin-binding proteins and EF-hand containing proteins in the present study, it would be acceptable that the HR calcium-related genes take an active part in heat response in rice panicle through perception and transduction of calcium signal.

Small GTPases are GTP-binding proteins [97], and include Rho, Rab, Arf, and Ran families in plant. RAC/ROP GTPases belonging to Rho-like small GTPases function in auxin-regulated gene expression and directional auxin transport [98], and play multiple roles in diverse cellular activities including ROS production and cell death in rice [99]. The *Arabidopsis* RabE enhanced plant defenses [100]. Overexpression of *TaRAN1* in wheat demonstrated that Ran has vital roles in auxin signaling meristem initiation [101]. The activation of the small GTPases requires a GTPase-activating protein (GAP). A putative ArfGAP gene in *Arabidopsis* is specifically expressed in roots, pollen grains, and pollen tubes [102]. The guanine nucleotide dissociation inhibitor (GDI) negatively regulated the GTPases activity [103]. In our case, the 9 HR small GTPases genes were early or continuously increased during heat stress, and only the 4 genes for Rab11D, RacGAP, Arf and ArfGAP were repressed (Table S8). Therefore, the small GTPase family genes may play very important roles in response to heat shock in rice panicle and they exhibited sophisticated and antagonistic functions to maintain cellular balance.

### Cross-talk in Heat Stress Response

**Figure 9:**
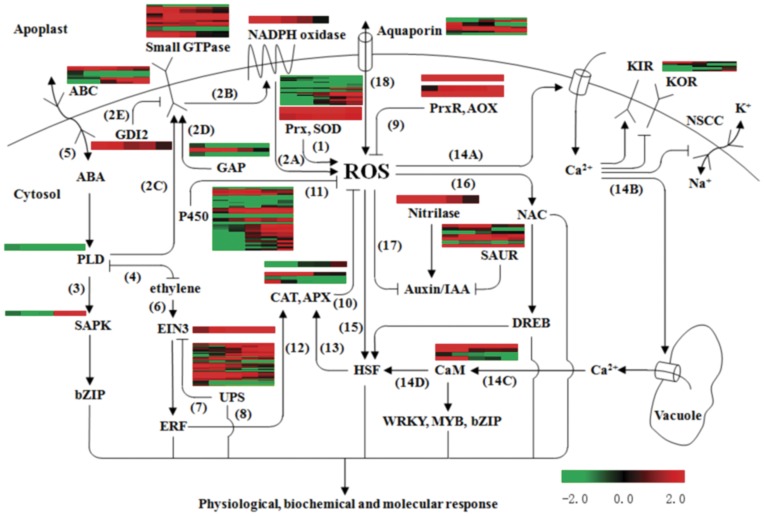
Regulation model central to ROS. The catalysis by Prx, SOD and NADPH oxidase (also called Rboh) lead to ROS accumulation (1, 2A,), and Rboh activity is controlled by the small GTPase (2B) that were positively regulated with PLD-derived PA (2C) and GAP proteins (2D), and negatively regulated by GDI2 (2E). PLD is a component of ABA signaling pathway to activate SAPK (3) and is the key point at which ethylene and ABA showed negative interplay (4). ABA can be diffused through ABC transporters (5). In ethylene signaling pathway, EIN3 activates ERF genes (6), and was degraded by the UPS (7). The UPS reglated plant growth and stress tolerance (8). ROS could be abolished by PrxR, AOX (9), CAT, APX (10) and P450 (11). The CAT and APX genes were trans-activated by ERF (12) and HSF (13). ROS activate calcium influx channel (14A), and stimulates Ca^2+^ release from internal Ca^2+^ stores (14B). Ca^2+^ stimulates CaM (14C), which activates HSF activity (14D). ROS stimulate HSF (15) and NAC (16). ROS exert negative regulation on auxin signaling pathway that is suppressed by SAUR and stimulated by nitrilase, respectively (17). Furthermore, ROS could be diffused through aquaporin (18). The HR genes in the present study were mapped on this model. The color bar represents the log2 transformed relative expression level on the time course of heat treatment (from left to right: 20 min, 60 min, 2 h, 4 h, and 8 h in sequence). The color scale is shown at the right. Blue: down-regulation, red: up-regulation.

It’s widely known that ROS generation is a general plant-cell response to various stresses. Models for roles of ROS in stress responses [104], [105] as well as in GA and ABA signaling in barley aleurone cells [106] had previously been proposed and discussed. Based on these previous work and our microarray data, we have proposed a regulation model central to ROS to pinpoint the cross-talk in response to heat stress in rice panicle (Fig. 9).

ROS production is promoted by series of enzymes such as Prx, SOD, and NADPH oxidase. Prx mediated H_2_O_2_ synthesis that causes stomatal closure in *Vicia faba* epidermal strips under UV-B radiation [107]. The membrane-bound respiratory burst oxidase homologue (Rboh)-NADPH oxidase can be enhanced by the small GTPase that are activated through PLD-derived PA [108]. PLD stimulates ABA signaling by activating SAPK and downstream components [87]. And ethylene negatively regulates ABA signaling through inhibiting PLD activity, and reciprocally, PA directly regulates ethylene signaling [108]. In addition, the direct binding of Rac1 of small GTPase to RbohB in rice leads to an increase in ROS [109].

It’s well known that CAT, APX, PrxR, AOX and P450 family genes lead to ROS clearance. CAT and APX were activated by SUB1A, an ERF transcription factor, resulting in enhanced tolerance to drought stress [110], and ERF genes were regulated through EIN3 in ethylene signaling [111]. EIN3 was found to be targeted and eliminated by the UPS [89].

ROS are diffused through aquaporin to amplify their effects [112], and activate plasma membrane Ca^2+^-permeable channels, resulting in an increase in cytoplasmic Ca^2+^, which further stimulates Ca^2+^ release from internal Ca^2+^ stores [113]. ROS regulate Na^+^/K^+^ homeostasis through elevating cytosolic Ca^2+^ levels followed by inhibition of NSCC (non-selective cation channels) and KOR (the outward-rectifying K^+^ channels), and activation of the inward-rectifying K^+^ channels (KIRs) in plants [114]. Besides, ROS signature can be perceived by CaM [115], NAC family proteins [116] and HSFs [117].

The HR genes in the present study were mapped on this model (Fig. 9). Our analysis showed that the genes for ROS generation were induced under high temperature, such as SOD, NADPH oxidase and the small GTPase genes. However, the genes encoding PLD and GAP were largely down-regulated, and GDI2 was greatly elevated, which implied that there is a fine mechanism to regulate the small GTPase activity. At the same time, SUB1A gene that is absent in rice microarray and can activate CAT and APX gene expression was early induced by heat shock in rice panicle by qRT-PCR (data not shown). However, the genes for CAT and APX were down-regulated. PrxR and AOX genes were significantly induced, and 10 P450 family genes were up-regulated at the late stage of heat stress to repress ROS burst. It suggested the possible complicated mechanism to maintain ROS homeostasis. Moreover, the data showed that auxin, ethylene and ABA signaling pathways were tightly regulated. The aquaporin and ABC genes were induced to some extent, which facilitates ROS and ABA spread though these channels to magnify their effects. Taken together, our data revealed complex cross-talk and the importance of ROS balance in response to heat stress.

**Figure 10:**
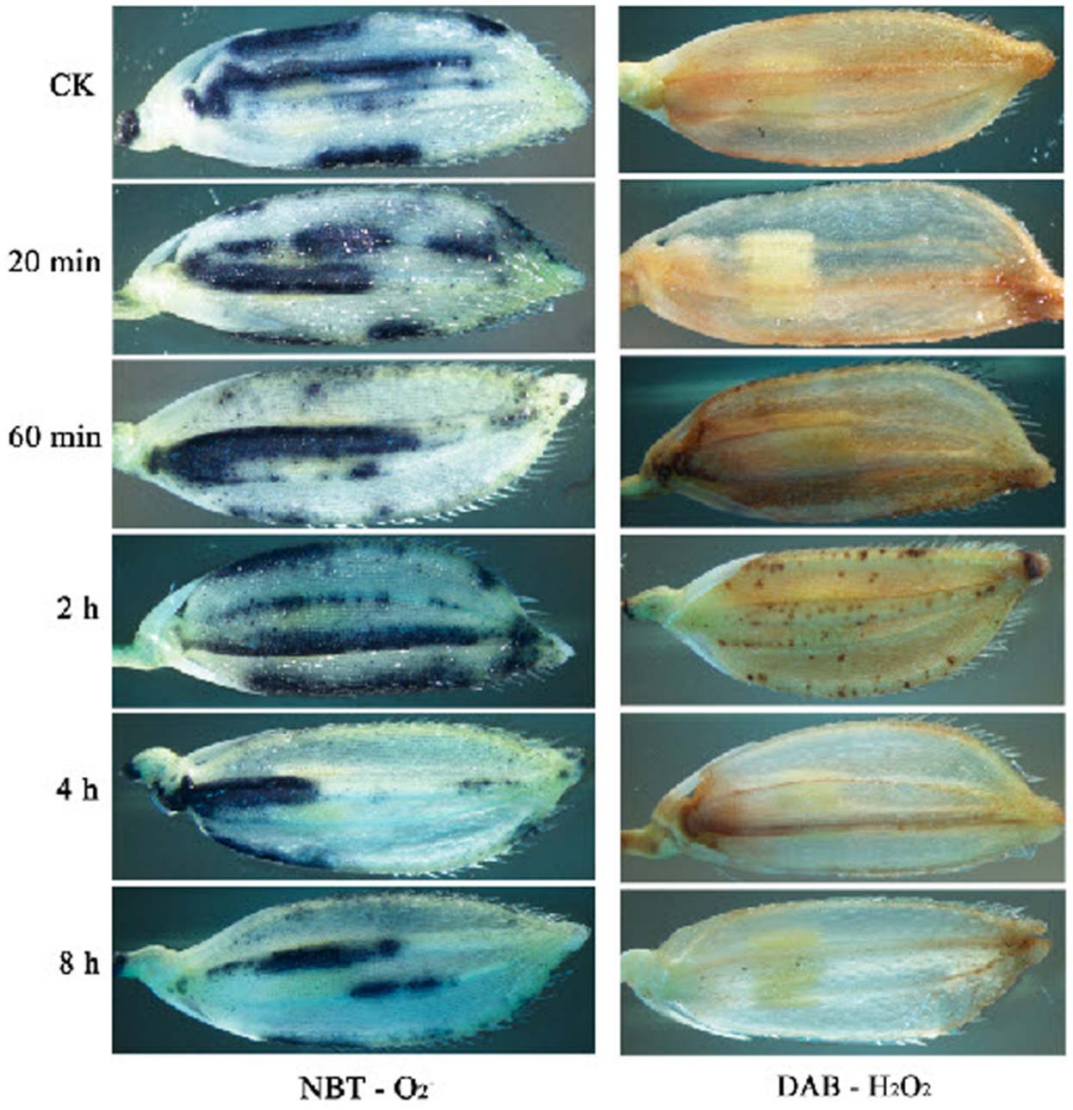
Accumulation of superoxide anion and hydrogen peroxide in rice panicle under heat shock. Plants at the reproductive stage were treated at 40°C for 8 h, and the florets of young panicles were collected at 0 min (as a control), 20 min, 60 min, 2 h, 4 h and 8 h. Immediately following heat-treatment, the florets were treated with NBT solution to detect superoxide anion or DAB solution to detect hydrogen peroxide in the dark for 3 h and 24 h, respectively.

To obtain the concrete evidence for this model, we detected the dynamic characters of the major ROS, superoxide anion and hydrogen peroxide, during heat treatment in rice panicle using NBT and DAB staining, respectively. The results showed that ROS were characteristic of the immediate accumulation upon heat shock and decrease at the late stage of heat shock compared with the untreated samples (Fig. 10, Table 1), which is basically consistent with previous work [4]. The dynamic changes of superoxide anion and hydrogen peroxide in rice panicle supported the hypothesis that ROS balance is pivotal for rice panicle survival under heat stress.

**Table 1:** Detection and quantification of superoxide anion and hydrogen peroxide in rice panicle under heat shock.

ROS	CK	20 min	60 min	2 h	4 h	8 h
NBT - O2-	++	++	++	+++	+	+
DAB - H2O2	++	++	+++	+++	+	+

Note: ++ represent for the normal level of superoxide anion and hydrogen peroxide in rice panicle, +++ for the high accumulation, + for the low level.

Furthermore, the promoter analysis on co-regulated genes provided more evidence for the cross-talk during heat treatment. It indicated that, among 12 clusters of KMC, GCC box, CE3, ABRE and HSE were significantly enriched only in the early up-regulated Cluster 9, and GCC box and CE3 were the centers of two co-occurrence networks, respectively (Fig. 7), which implies their important roles in heat response. Earlier study elucidated the function of GCC box in ethylene-induced gene expression [118]–[120]. Recently, OsDERF1, a novel ERF transcriptional activator, was found that binds to GCC box to activate the expression of repressors, subsequently suppress ethylene synthesis and reduce drought tolerance in rice [121]. Moreover, CE3 and ABRE were proved to be essential and sufficient for ABA signaling and other stimuli such as Ca^2+^ and drought [122]–[124]. It’s also widely known that HSE can be recognized and bound by activated HSF to respond to cellular and environmental changes, especially to heat shock [125]–[127]. Our data showed that ethylene signaling involved in GCC box and ABA signaling with CE3 plus ABRE may have indispensable roles in response to heat. In addition, the outstanding function of HSE should be highlighted due to its close relationship with CE3 and GCC box in the co-occurrence network, which indicates the complexity and wide existence of cross-talk during heat shock.

## Materials and Methods

### Plant Materials and Heat Treatments

Heat-tolerant rice cultivar 996 was cultivated in the university experimental rice field until reaching at the anther development stage 8 as defined by Zhang and Wilson [128], and then was moved into a growth chamber (Binder, Tuttlingen, Germany). The plants were pretreated under the condition of 32°C/28°C (day/night) with 80% humidity for one day (12 h/12 h) in the growth chamber, and then the control samples were collected before continuous heat treatment at 40°C with 80% relative humidity and illumination intensity of 600 µmol m^−2^ s^−1^. Young florets at the anther development stage 8 at the middle of main panicles were collected at the time points of 0 min, 20 min, 60 min, 2 h, 4 h, and 8 h after heat treatment, frozen in liquid nitrogen immediately, and stored at −80°C for hybridization.

### RNA Isolation, Microarray Hybridization, Signal Scanning and Normalization

Total RNA was isolated using Trizol (Invitrogen, Carlsbad, CA), and further purified using QIAGEN RNeasy kit (Qiagen Valencia, CA) following manufacturer’s specifications. Agilent 4×44K rice oligo microarray with 42489 60-mer oligonucleotide probes was used for expression profile analysis. Twelve microarrays of single dye with two independent replicates for each time point were performed according to the manufacturer’s protocol.

The above hybridization signal data files in text format from the Feature Extraction Software were imported into GeneSpring GX (Agilent Technologies). The detected, not detected and compromised data were marked with specific flag of P, A, M, respectively. The data were normalized by Quantile algorithm followed by the process of baseline to median of all samples. The normalized data were log2 transformed, and the correlation coefficient of replicates was determined by hierarchical clustering using complete linkage algorithm as implemented TIGR MeV version 4.0 [129], [130].

### Data Filtering, Clustering and Functional Classification

Data filtering was conducted with the criteria that at least 6 out of 12 samples have P flag to ensure the quality of the normalized data. The average expression value of each time point was used to get the ratio of the expression change between the treatment and un-treatment samples, and obtain the differentially expressed genes with 3-fold change at each time point of heat treatment, and the Venn diagram analysis was carried out to see the significantly regulated genes by heat. The heat-responsive genes were screened if the ratio of expression level has 3-fold change or greater in at least two time points on the time course, HCL and KMC incorporated in Mev were performed to get co-expression pattern. The gene classification based on gene ontology was conducted as described by Hobo et al. [131]. The pathway analysis was performed with MapMan [132] after converting RAP_ID into TIGR_ID according to the annotation from RAP_DB (http://rapdb.dna.affrc.go.jp/).

### Analysis on Promoters of Co-expressed Genes

To better understand the gene expression regulation in heat response, promoters of co-expressed genes from the 12 clusters of KMC were analyzed. The known *cis*-acting elements was extracted from PlantCARE database [133]. The motifs were scanned by FIMO [134] in the −3000 bp upstream ATG of all genes in rice genome. The output results were stored in a local database as background for further analysis. The enrichment ratio of the motif in a promoter was defined as:
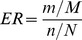



Here, m is the number of genes containing the specific motif in the individual cluster. M is the gene number of specific cluster. And n is the number of genes containing the specific motif in our database. N is the gene number in the database.

The enrichment of a motif in a cluster was assessed by the hypergeometric distribution. The p-value for a motif in the cluster was defined as:
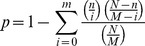



The motif was considered as enriched in the specific cluster when p<0.05.

Finally, motif co-occurrence analysis was performed to find the relationship of cis-acting elements under heat stress in rice panicle. When two motifs were found within 250 bp in a promoter, it was considered as co-occurring once. When the two motifs co-occur multiple times in one promoter, they are only counted as one co-occurrence. The significance of co-occurrence was assessed by the hypergeometric distribution [135]:
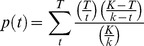



K and T are the gene numbers in the database and in the individual cluster, respectively. The k and t are the numbers of genes containing the specific motif in the database and in the individual cluster, respectively. The adjacent two motifs in the specific cluster were considered as significantly co-occurring when p<0.05.

The number of genes containing the specific motif, enrichment ratio for a single motif and p-value of motifs co-occurrence were imported into Cytoscape [136] as the node size, the color of nodes, and the shape of nodes, respectively.

### qRT-PCR Analysis

To validate microarray results, 10 genes expression with significant expression changes in the microarray data was verified by qRT-PCR. Among the multiple probes, only one probe with the most significant expression data was chosen and its fold change was calculated by comparing the average data at each time point of heat stress with that of the control. Gene specific oligonucleotide primers were designed for amplicons about 200 bp for each gene (Table S10) using Primer Premier 6.0. A rice *actin1* gene [137] was used as an internal control in each reaction.

The same RNA samples for microarray hybridization were used for qRT-PCR. Prior to synthesis of cDNA, any residual genomic DNA was removed by DNase I treatment (Invitrogen, Carlsbad, CA) according to the manufacturer’s instructions. First-strand cDNA was synthesized with Oligo (dT) primer using SuperScript III RT (Toyobo, Osaka, Japan).

Polymerase chain reactions were carried out in 96-well in the iCycler iQ5 (Bio-Rad, Hercules, CA) using SYBR Green I PCR Master Mix (Bio-Rad). Each 25 µL real-time PCR reaction mixture containing 10.5 µL dd-water, 0.5 µL 200 nM each of forward and reverse primers, 12.5 µL of SYBR Green I Master, and 100 ng of cDNA. PCR reaction conditions were as follows: 95°C for 20 s, and followed by 40 cycles of 95°C for 5 s, 60°C for 20 s and 72°C for 31 s.

A negative control without cDNA template was run with each analysis to evaluate the overall specificity. For each gene, three technical replicates and two biological replicates were used at each sampling time point. The quantification of gene expression was performed using the relative quantification method (2^−ΔΔCT^) [138] and comparing data with internal control.

### Detection and Quantification of ROS

ROS accumulation in rice panicle under heat shock was detected according to Fukao et al [139]. Plants grown under normal condition were used as a control. All samples were collected at 0 min, 20 min, 60 min, 2 h, 4 h and 8 h after heat treatment for ROS detection, respectively. To visualize superoxide accumulation, the young florets at the middle of main panicles of heat-treated or untreated control rice were excised and immediately placed in a 0.5 mg/mL NBT solution in 10 mM potassium phosphate buffer, pH 7.6, at 25°C for 3 h in the dark. For hydrogen peroxide detection, the young florets were treated with 1 mg/mL DAB in 50 mM Tris acetate buffer, pH 5.0, at 25°C for 24 h in the dark. Each experiment was repeated on at least three different plants, and representative images are shown.

### Data Deposition

Our microarray data were deposited in the Gene Expression Omnibus under accession number GSE38665.

## Supporting Information

Table S1 **Correlation coefficient of microarray replicates.**
(XLS)

Table S2 **The significantly regulated genes on the time course.**
(XLS)

Table S3 **The HR genes on the time course of heat stress.**
(XLSX)

Table S4 **The HR transcription factors.**
(XLS)

Table S5 **The HR Hsp and other chaperone genes.**
(XLSX)

Table S6 **The HR transporter genes.**
(XLS)

Table S7 **The HR ROS-related genes.**
(XLSX)

Table S8 **The HR signaling-related genes.**
(XLSX)

Table S9 **The HR ubiquitin-proteasom system genes.**
(XLSX)

Table S10 **qRT-PCR primer sequence.**
(XLSX)
